# Bulk RNA-seq datasets analysis integration identifies robust drought-responsive genes and functional networks in *Eucalyptus grandis*

**DOI:** 10.3389/fbinf.2026.1743474

**Published:** 2026-04-10

**Authors:** João Vítor Aires-Teixeira, Nivea Maria Pereira Lima, Renato Almeida Sarmento, Gabriel Quintanilha-Peixoto, Kellen Kauanne Pimenta de Oliveira

**Affiliations:** 1 Laboratory of Molecular and Functional Genetics (LAGEMF), Bioprocesses and Biotechnology Engineering, Federal University of Tocantins (UFT), Gurupi, Brazil; 2 Federal University of Tocantins (UFT), Palmas, Brazil; 3 Laboratory of Chemistry and Function of Peptides and Proteins, Center of Biosciences and Biotechnology, State University of Norte Fluminense (UENF), Campos, Brazil

**Keywords:** abiotic stress, climate-resilient crops, eucalyptus, gene networks, *in silico*, meta-analysis, systems biology, transcriptomics

## Abstract

**Introduction:**

*Eucalyptus grandis* is a cornerstone of global forestry, yet its productivity is increasingly threatened by drought. Understanding the molecular mechanisms underlying drought response is essential for improving resilience.

**Methods:**

We conducted a meta-analysis of three independent RNA-Seq drought studies in *E. grandis*, applying a rigorous bioinformatic pipeline with leave-one-out Jackknife validation to ensure robustness and reduce single-study bias.

**Results:**

We identified a high-confidence set of 472 differentially expressed genes (DEGs), including 274 upregulated and 198 downregulated genes. Functional analysis revealed a growth-defense tradeoff, with upregulated genes associated with stress response pathways such as protein folding, osmolyte biosynthesis, and ABA signaling, while genes involved in cell division, DNA replication, and cell wall biosynthesis were repressed. Protein–protein interaction analysis showed a coordinated network linking stress response activation to suppression of growth-related processes.

**Discussion:**

These findings provide a robust catalog of candidate genes, including previously uncharacterized proteins, supporting future functional studies and molecular breeding strategies aimed at enhancing drought tolerance in eucalyptus under climate change.

## 1 Introduction

The escalating climate crisis presents unprecedented challenges to global ecosystems and forestry, with projections indicating continued increases in temperature and the frequency of extreme weather events such as droughts. Since pre-industrial times, the Earth’s surface temperature has risen by 1.1 °C and could surpass 4 °C by the end of this century. Plant populations existing outside their optimal climate conditions are likely to experience widespread die-offs, leading to reductions in their geographic distribution, a trend that poses a risk to one in every six species worldwide (IPCC, 2023).

The existing literature describes that plants have evolved complex molecular and physiological responses to cope with such stresses, including osmotic adjustment, antioxidant defense, and the modulation of growth and development, often orchestrated by intricate networks of regulatory genes (Benešová et al., 2012; Zhang et al., 2022; Quintao et al., 2023; Khan, 2025). The Myrtaceae family, to which eucalyptus belongs, encompasses a wide range of species adapted to diverse environments, offering a rich genetic resource for understanding stress tolerance mechanisms; however, the specific responses of key commercial species like *Eucalyptus grandis* to intensifying drought require deeper investigation (Souza et al., 2023).

*E. grandis* is a pillar of the global forestry economy, primarily due to its rapid growth and versatile wood, which is extensively used in pulp, paper, timber, and bioenergy production (de Medeiros et al., 2024). Brazil stands as one of the world’s leading producers of eucalyptus, along with India, China, and Australia, with extensive cultivated areas significantly contributing to its gross domestic product and export revenue, particularly through the pulp, paper, and bioenergy industries (Gustavsson et al., 2017; Cunha et al., 2021). While these cultivations are widespread, their expansion and sustainability, particularly in regions transitioning or bordering sensitive biomes like the Brazilian Cerrado, face increasing environmental scrutiny and the need for adaptive management practices, especially concerning water use and climatic resilience (dos Santos and Reichert, 2022; Silva et al., 2024). The Cerrado, a global biodiversity hotspot, faces significant anthropogenic pressures, especially due to overfarming and agricultural land expansion (Colman et al., 2021). In this context, the investigation and improvement of drought-tolerant tree species like *E. grandis* becomes crucial for both economic and ecological perspectives (Bacani et al., 2024).

Among possible strategies for *E. grandis* improvement, RNA sequencing (RNA-Seq) represents a powerful and widely adopted tool for comprehensive gene expression analysis (transcriptomics) (Tyagi et al., 2022). Various studies have used this approach to quantify gene expression levels, identify novel transcripts, and alternative splicing events in plant species, offering deep insights into the dynamic molecular responses of organisms to various stimuli, including abiotic stresses like drought (Safavi-Rizi et al., 2020; Liu et al., 2021; Rutley et al., 2021; Reddy et al., 2013; Zhang et al., 2022; Yan et al., 2024; Zhu et al., 2024; Yu et al., 2025).

In this context, we performed a meta-analysis integrating data from multiple independent RNA-Seq studies of *E. grandis* to assess the consistency of findings related to drought response (Kukurba and Montgomery, 2015). This approach allows us to overcome the limitations inherent in individual experiments, such as small sample sizes or specific conditions, thereby addressing a critical gap in the literature regarding a comprehensive view of drought response. By increasing the statistical power, we present a reliable and comprehensive catalog of essential genes and pathways for developing targeted strategies to enhance drought resilience in this economically important species.

## 2 Methods

### 2.1 RNA-seq datasets

Three independent RNA-seq studies were selected from the NCBI SRA database (available at https://www.ncbi.nlm.nih.gov/sra, accessed on March 2026), adhering to strict inclusion criteria: paired-end Illumina data from *E. grandis* experiments comparing exclusively well-watered controls against drought-stressed samples. Dataset selection followed strict inclusion criteria designed to balance biological generality with technical consistency: (1) paired-end Illumina sequencing (platform consistency), (2) *E. grandis* as focal species (genetic comparability), (3) ≥ 3 biological replicates per condition (statistical power), (4) secondary vascular tissue sampling (biological relevance), (5) controlled or semi-controlled drought experiments (experimental rigor), (6) documented physiological stress validation (biological verification), and (7) publicly available raw sequencing data in SRA. Complete metadata for selected studies are provided in Supplementary File S1.

Raw sequencing data were retrieved via in-house bash scripts. The script used the fasterq-dump module of SRA Toolkit v3.2.1 (Leinonen et al., 2011) for data download, with GNU Parallel 20250422 (Tange, 2018) to execute simultaneous download and processing, accelerating the retrieval of all samples. Following the download, paired-end FASTQ files were compressed and renamed according to a predefined mapping scheme to ensure consistency. The entire process featured comprehensive logging and error handling to guarantee data integrity.

### 2.2 Quality control

Quality control was implemented using FastQC v0.12.1 (Andrews, 2023). The reports were aggregated using MultiQC v1.28 (Ewels et al., 2016). Adapter trimming and read filtering were performed with bbduk from BBTools v39.23 (Bushnell et al., 2017), applying parameters including k-mer trimming (k = 23, mink = 11, hdist = 1), quality-based trimming (Q ≥ 10), and removal of reads shorter than 50 bp. Paired-end consistency was enforced (tpe, tbo flags).

### 2.3 Salmon quantification

Transcript quantification was executed with Salmon v1.10.3 (Patro et al., 2017) in alignment-based mode, correcting for GC content and sequence-specific biases. The index was constructed using the *E. grandis* reference transcriptome (GenBank accession code GCF_016545825.1). Salmon output files were organized into study-specific directories matching the original SRA project identifiers for systematic downstream analysis.

### 2.4 Differential gene expression and meta-analysis integration

Adapting the approach of Hosseini et al. (2023) (Hosseini et al., 2023) work, transcript-level quantifications were imported into R v4.4.3 using tximport v1.34.0 (Soneson et al., 2015). Gene-level counts were then filtered for low expression using the filterByExpr module of edgeR v4.4.2 (Chen et al., 2025), and libraries were normalized using the Trimmed Mean of M-values (TMM) method. Subsequently, differential gene expression analysis was performed independently for each of the three studies using DESeq2 (v1.46.0) (Love et al., 2014). To ensure stability, log2 fold changes (LFCs) were moderated using the lfcShrink function with the ashr estimator. Meta-analysis was then conducted on genes common across all studies. Raw p-values from individual analyses were combined using Fisher’s method metaRNASeq v1.0.8 (Marot et al., 2025), and a combined effect size was calculated as a weighted average LFC. Significant meta-DEGs were defined as genes with a combined FDR <0.05 and an absolute weighted LFC ≥ 1. The stability of these findings was assessed using a leave-one-out Jackknife resampling procedure; genes that remained significant throughout the full analysis and all Jackknife iterations were termed as robust DEGs.

Finally, a highly rigorous common DEGs set was identified by further filtering these DEGs, requiring them to also be individually significant in all three studies (FDR <0.05 , |LFC|≥1 ) with a consistent direction of regulation. To evaluate the stability of the results and the influence of each dataset on the meta-analysis, the Jackknife validation was detailed through a quantitative analysis. The Jaccard dissimilarity was calculated to measure the magnitude of change in the DEGs set upon the exclusion of each study. In addition to this index, the impact of each study was characterized by quantifying gene flux: “genes lost” (DEGs that ceased to be significant after a study’s exclusion) and “genes gained” (genes that became significant in its absence) were tallied. This dual approach allowed measuring the magnitude of the change (dissimilarity), providing a more robust assessment of each study’s contribution to the final meta-analysis result. The entire process was implemented in the R environment with custom scripts. Resulting plots were generated with ggplot2 v3.5.2 (Wickham, 2016) and ggVennDiagram v1.5.2 (Gao and Dusa, 2024). Data manipulation was handled with the tidyverse suite v2.0.0 (Wickham and RStudio, 2023).

### 2.5 Functional enrichment and network analysis

To build the protein-protein interaction (PPI) networks, protein sequences of the consistently up- and downregulated genes were retrieved for *E. grandis* (egr) via KEGG REST API. The PPI networks were then assembled using the STRING database v11.30 (Szklarczyk et al., 2023), with *E. grandis* as the reference organism. A medium confidence cutoff of 0.400 was adopted, following established thresholds for plant interactomes. Functional enrichment analysis was performed using STRING’s built-in tools, with Gene Ontology (GO) terms clustered by semantic similarity (threshold ≤ 0.8) and sorted by gene count to prioritize biologically coherent pathways. The networks were visualized and annotated in Cytoscape v3.10.3 (Shannon et al., 2003), integrating mean Fold Change (FC) values and node-specific enriched GO terms for enhanced interpretability, considering an FDR ≤ 0.004. All graphs were annotated and personalized with Inkscape v1.4. Protein annotations and fold-changes for network nodes are provided in Supplementary File S2.

All Salmon quantification files for each analyzed sample, the complete R scripts used for the meta-analysis pipeline, and the processed outputs, including robust DEG lists with corresponding meta-analytical statistics, are publicly available in the project repository, available at https://github.com/jvtarss/euca2025, which contains the quantification data, analysis scripts, and final results.

## 3 Results

The RNA sequencing datasets of three independent studies, designated Study A (SRP178354), Study B (SRP458400), and Study C (SRP406292), were processed to assess data quality and quantify gene expression, as shown in Table 1. These datasets represent complementary experimental designs spanning diverse biological contexts. Study A (Ployet et al., 2019) employed a field trial with 4-year-old trees subjected to chronic drought (90 days), with documented predawn water potential ( $\Psi_{\text{pd}}$ : −1.8 MPa under drought vs. −0.3 MPa in controls) and relative water content (65% vs. 95%), sampling secondary xylem. Meanwhile, Study B (Keret et al., 2024) used controlled growth chambers with 6-month-old saplings exposed to acute drought (30 days), with physiological monitoring of stomatal conductance (25 vs. $120\;mmol\;m^{-2}\;s^{-1}$ ) and soil water content (0.065 vs. $0.283\;m^{3}\;m^{-3}$ ), sampling stem xylem. Finally, Study C (Teshome et al., 2023) employed greenhouse conditions with 2-year-old seedlings under acute drought (17 days), sampling whole stem tissue. While tissues differ in anatomical specificity, all focused on secondary vascular tissue, the primary site of drought-responsive wood formation. Study A produced an average of 22.27±4.60 million reads per sample, while Study B averaged 29.31±3.11 million reads, and Study C produced the highest average count at 81.13±12.91 million reads. The transcript alignment rates using Salmon were consistently high across all studies, with Study A showing 89.35%±0.96% , Study B 88.28%±0.86% , and Study C 88.22%±0.57% mean alignment percentages. Following transcript-to-gene mapping and initial filtering for low expression, a total of 26,821 genes were retained for downstream differential expression analysis.

**TABLE 1:** Number of reads, alignment rate per sample, and number of DEGs per study, before and after Jackknife analysis.

Parameter	Study A	Study B	Study C
	(SRP178354)	(SRP458400)	(SRP406292)
Reads (millions ± SD)	22.27±4.60	29.31±3.11	81.13±12.91
Alignment (% ± SD)	89.35±0.96	88.28±0.86	88.22±0.57
Individual DEGs (up, down)	571 (365, 206)	2783 (1044, 1739)	909 (482, 427)
Jackknife exclusion DEGs (up, down)	3214 (1244, 1970)	1302 (722, 580)	3005 (1242, 1763)

**FIGURE 1:**
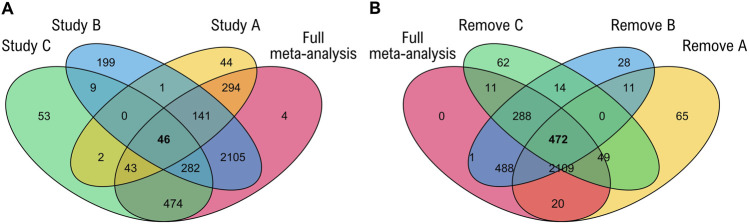
Venn diagrams showing **(A)** the number of common DEGs shared among the individual studies and meta-analysis, and **(B)** the number of common DEGs among different Jackknife tests and meta-analysis results.

Figure 1A revealed a core set of 46 common genes identified by all three individual analyses and 133 genes identified exclusively by the overall meta-analysis when comparing the DEGs from each study with the significant Meta-DEGs. The Jackknife resampling procedure culminated in the identification of a highly robust core set of DEGs; as depicted in Figure 1B, a total of 472 genes consistently met the significance criteria (meta-analysis FDR <0.05 and |weighted LFC|≥1 ) across the full meta-analysis and all three leave-one-out Jackknife iterations. Within this robust set, 274 genes were consistently upregulated, and 198 were consistently downregulated.

**FIGURE 2:**
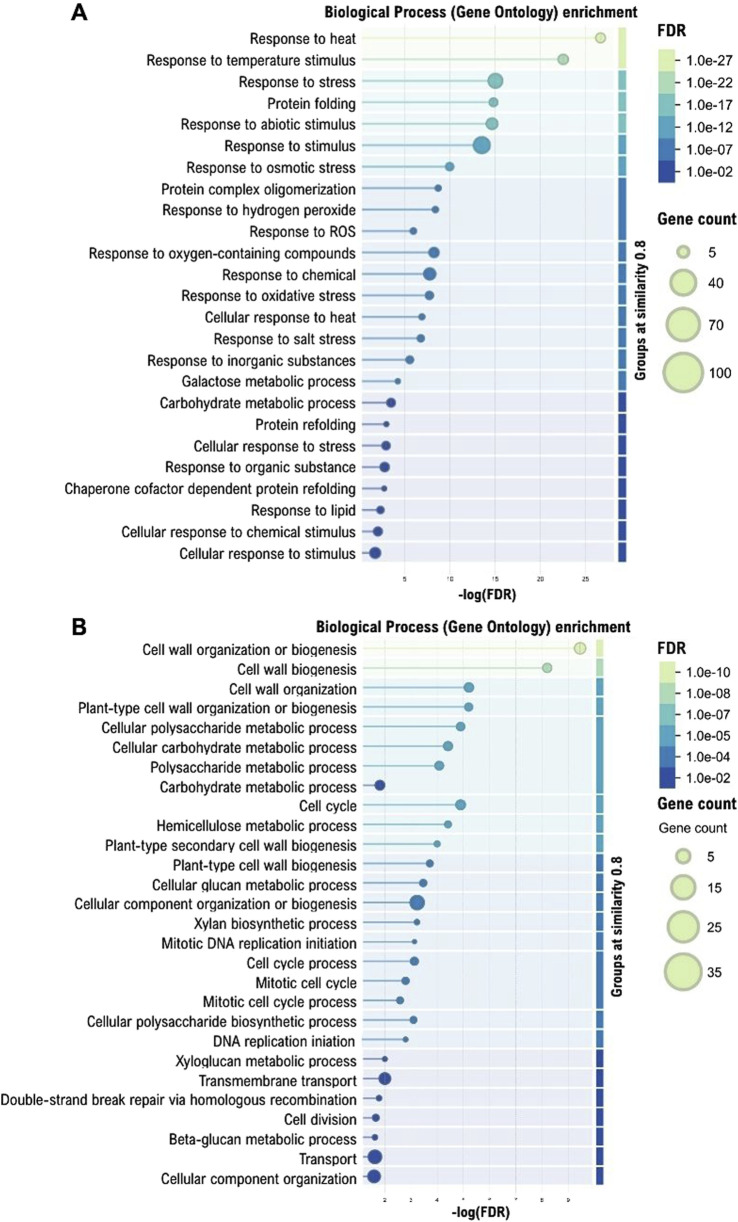
Gene Ontology (GO) enrichment analysis of Biological Processes (BP) for differentially expressed genes, with term networks generated by STRING. **(A)** Enriched GO BP terms for upregulated genes. The displayed terms have False Discovery Rates (FDR) ranging from $1\times 10^{-27}$ to $1\times 10^{-2}$ . **(B)** Enriched GO BP terms for downregulated genes, with displayed terms having FDRs in the range of $1\times 10^{-10}<FDR<1\times 10^{-2}$ . In both panels, significance is visualized on a −log(FDR) scale, and terms were functionally grouped based on a similarity coefficient of 0.8.

We performed a Gene Ontology (GO) enrichment analysis of the Meta-DEGs; the upregulated genes (Figure 2A) included highly significant terms such as response to heat, protein folding, response to abiotic stimulus, and stress response. Meanwhile, for the downregulated genes (Figure 2B), enriched GO terms were predominantly associated with cell wall organization or biogenesis, cell cycle, mitotic cell cycle, and polysaccharide metabolic processes related to cell wall composition.

**FIGURE 3:**
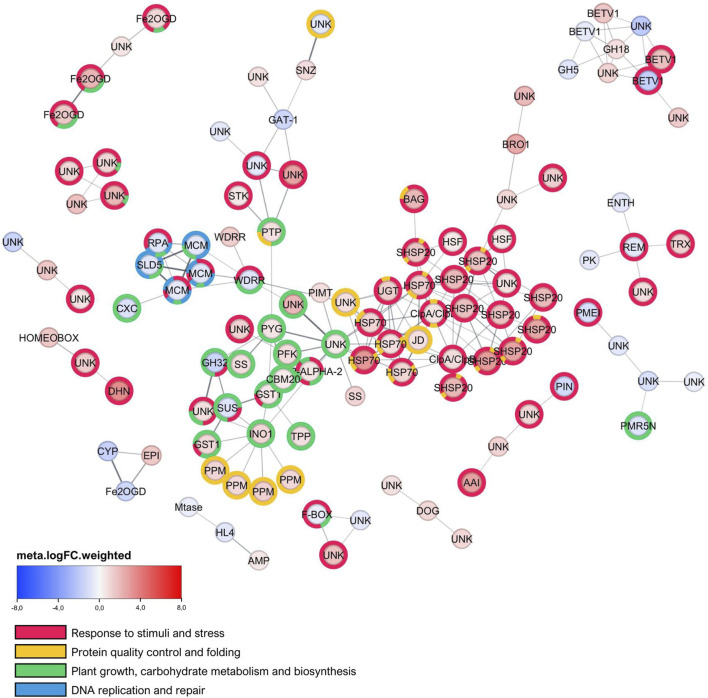
PPI network of differentially expressed genes (DEGs), generated using STRING and visualized with Cytoscape. The network includes both up- and downregulated DEGs. Unique isolated nodes and clusters containing only two nodes were removed. Nodes are annotated with Gene Ontology (GO) terms using a flat donut label style, with terms grouped into broader categories. The GO terms displayed have False Discovery Rates (FDR) in the range of $4.17\times 10^{-17}<FDR<4.15\times 10^{-2}$ . The network was organized using the Prefuse Force Directed layout algorithm, weighted by STRING interaction scores.

The Protein-Protein Interaction (PPI) network (Figure 3) revealed a prominent and highly interconnected cluster of upregulated proteins. This central group included heat shock proteins (SHSP20, HSP70), ClpA/ClpB proteases, and proteins involved in osmolyte synthesis (INO1, TPP). The network also indicated downregulation of proteins essential for DNA replication (MCM, SLD5). Additional distinct clusters highlighted proteins involved in antioxidant defense (TRX), cell wall dynamics (PMEI), and hormonal signaling. Numerous uncharacterized proteins exhibited substantial expression changes within these functional groups.

## 4 Discussion

Climate projections indicate that the frequency and severity of drought events will intensify across tropical and subtropical regions where Eucalyptus farming is economically critical (IPCC, 2023). Extended dry seasons already reduce eucalyptus productivity by 20%–40% in major producing regions (Booth, 2013). The forestry industry urgently requires drought-tolerant cultivars, yet breeding programs have been hindered by incomplete knowledge of molecular mechanisms underlying drought adaptation in Eucalyptus secondary vascular tissues, where water transport capacity and wood quality traits are determined.

Individual transcriptomic studies have identified drought-responsive genes in Eucalyptus Ployet et al. (2019); Keret et al. (2024); Teshome et al. (2023), yet their limited overlap (only 46 genes significant across all three, Figure 1A) highlights the context-dependency of single-study findings. Our meta-analysis offers an improved approach by identifying signals robust to experimental heterogeneity. Here, we integrated three independent datasets to identify 472 genes whose drought-responsive expression is conserved across diverse genotypes, developmental stages, and stress protocols.

### 4.1 Methodological robustness and the core drought-responsive gene set

This meta-analysis enabled a more comprehensive characterization of the drought response in *E. grandis* than any single study alone, initially identifying a broad set of differentially expressed genes. The 472 robust genes represent 13.9% of the initial 3,394 meta-significant DEGs, a proportion consistent with stringent cross-study validation. For comparison, the sheep fat-tail RNA-Seq meta-analysis integrating six independent datasets which methodology was used as a major reference in the present work (Hosseini et al., 2023) identified 500 DEGs after directional filtering, of which 68 (13.6%) passed all six jackknife iterations, a retention rate strikingly similar to that observed here. Similarly, a meta-analysis of 216 paired RNA-Seq datasets spanning five ABA-related stress conditions in *Arabidopsis thaliana* (Shintani et al., 2023) retained only 22 genes (14 upregulated and 8 downregulated) consistently regulated across all treatment comparisons, reinforcing that stringent multi-study filtering systematically converges on a small but high-confidence gene core. Among individual *E. grandis* transcriptomic studies, (Keret et al., 2024), identified 3,048 DEGs under controlled drought conditions ( $|\log_{2}\text{FC}|>1$ , $p_{\text{adj}}<0.05$ ), yet cross-study overlap across the datasets included in our meta-analysis reached only 46 genes (0.98%). Our integration expanded this conserved core, demonstrating that meta-analysis substantially enhances the detection of reproducible drought-responsive signals in *E. grandis*.

This was followed by a rigorous validation of our findings using a Jackknife analysis. This procedure acted as a crucial filter to test the stability of the results. The analysis revealed that the exclusion of Study B (SRP458400) had a profound impact on the overall gene list, evidenced by a high Jaccard dissimilarity of 0.637. This value indicates a significant divergence, suggesting that Study B contributed a strong, unique transcriptional signature that was not consistently corroborated by the other datasets, which might stem from specific experimental conditions or genotypes.

### 4.2 Drought induces a growth-defense tradeoff in *Eucalyptus grandis*

Gene Ontology enrichment analysis revealed that the 472 robust genes are organized into functionally coherent modules rather than acting as independent stress responders. For the upregulated genes (Figure 2A), upregulated genes converged on stress protection pathways (response to heat: GO:0009408, FDR $<1\times 10^{-16}$ ; protein folding: GO:0006457, FDR $<1\times 10^{-14}$ ; response to osmotic stress: GO:0006970, FDR $<3\times 10^{-5}$ ). A strong enrichment of processes related to stress response and protein metabolism (especially folding) was observed. This aligns with contemporary understanding that plants under drought activate a complex network of protective mechanisms to maintain cellular homeostasis (Bacani et al., 2024). Highly significant terms included response to heat, response to temperature stimulus, protein folding, response to abiotic stimulus, and stress response. Drought frequently induces secondary oxidative and heat stresses, necessitating such multifaceted responses (Zandalinas and Mittler, 2022; Souza et al., 2023). These GO terms are not randomly distributed across the gene set; they map onto spatially distinct modules within the protein-protein interaction network, indicating that functional enrichment reflects coordinated regulatory programs rather than independent gene responses. The molecular basis for these observations in our eucalyptus analysis involves several key gene families whose roles in stress adaptation are well-documented in recent literature (Ployet et al., 2019; Teshome et al., 2023; Keret et al., 2024).

The significant upregulation of genes encoding small heat shock proteins (SHSP20), various chaperones of the ClpA/ClpB family, and numerous Heat Shock Protein 70 (HSP70) members is crucial. These molecular chaperones are essential for preventing protein misfolding and aggregation, and for refolding denatured proteins, thereby safeguarding cellular functions under stress. This role is consistently highlighted in plant stress physiology, including the studies on *E. grandis* referenced for this meta-analysis (Ployet et al., 2019), which also reported the induction of these protective gene families. Nonetheless, the authors further revealed the upregulation of genes involved in ROS detoxification, secondary metabolism, and the biosynthesis of protective compounds like polyamines and stachyose. Our results indicate the co-induction of Heat Shock Factors (HSF), which are master regulators of HSP expression, further emphasizing the activation of this chaperone-based defense (Wang et al., 2004; Kang et al., 2022). Additionally, BAG domain-containing proteins and J domain-containing proteins (JD), which function as co-chaperones that modulate HSP70 activity, were also prominent in our results, indicating a sophisticated regulation of protein quality control systems (Verma et al., 2019).

The upregulation of Fe(II)/2-oxoglutarate-dependent dioxygenases (Fe2OGD) is also significant. This vast enzyme superfamily is involved in critical plant processes, including the biosynthesis of hormones like abscisic acid (ABA), gibberellins, and ethylene, and the production of protective secondary metabolites such as flavonoids and other phenolics (Brookbank et al., 2021; Lange et al., 2023). Enhanced ABA levels, for instance, are pivotal for drought response, promoting stomatal closure and inducing wide-ranging transcriptional changes (Munemasa et al., 2015). Flavonoids, widely produced by eucalyptus, can act as potent antioxidants, mitigating oxidative damage, which is a common secondary effect of drought stress. The enrichment of non-specific serine/threonine protein kinases (STK) underscores the activation of crucial signaling pathways (Benešová et al., 2012; Wang et al., 2016). These kinases are key components in perceiving stress signals and transducing them to initiate downstream adaptive responses, including the regulation of gene expression and physiological adjustment (Lim et al., 2020).

Genes associated with maintaining genome integrity, such as DNA helicases (MCM) and Replication Protein A subunits (RPA), were also upregulated. Abiotic stresses like drought can lead to increased DNA damage, and DNA repair mechanisms are essential for plant survival and recovery (Aklilu et al., 2014; Mohapatra et al., 2023). The involvement of F-box domain-containing proteins, which are specificity factors for E3 ubiquitin ligases in the ubiquitin-proteasome system, points to the importance of regulated protein turnover. This system selectively degrades damaged or regulatory proteins, facilitating cellular reprogramming and resource reallocation under stress conditions (Wang et al., 2022). Finally, the enrichment of cellular carbohydrate metabolic processes, with genes like starch synthase (SS), trehalose-6-phosphate phosphatase (TPP), and phosphofructokinase (PFK), highlights metabolic reprogramming. Accumulation of soluble sugars such as sucrose, trehalose, raffinose family oligosaccharides is a common plant strategy during drought, serving as osmolytes for osmotic adjustment, osmoprotectants for cellular structures, and signaling molecules (Griffiths et al., 2025). Studies on eucalyptus species under drought have shown significant alterations in carbohydrate profiles, supporting their role in stress adaptation (Kong et al., 2021). Collectively, these upregulated gene categories in *E. grandis* reflect a comprehensive and evolutionarily conserved strategy to defend against drought, encompassing protein protection, detoxification, signaling, genome stability, and metabolic adjustments, all of which are actively researched areas in current plant stress biology.

Conversely, the downregulated genes (Figure 2B) revealed a significant suppression of processes primarily associated with cell growth, division, and cell wall biosynthesis. The enriched GO terms were dominated by categories such as Cell wall organization (GO:0071555) or biogenesis (GO:004254), Plant-type cell wall organization or biogenesis (GO:0071669), and various polysaccharide and carbohydrate metabolic processes linked to cell wall components (*e.g.*, Hemicellulose metabolic process (GO:0010410), Xylan biosynthetic process (GO:0045492), Xyloglucan metabolic process (GO:0010411), Beta-glucan metabolic process (GO:0051273). Furthermore, a clear downregulation of Cell cycle (GO:0007049), Mitotic cell cycle (GO:0000278) and DNA replication initiation (GO:000627) was evident. The suppression of Transmembrane transport (GO:0055085) and Cellular component organization (GO:0016043) also points towards a general reduction in cellular growth and expansion activities. This pattern suggests a strategy of resource conservation under drought, where energy and resources are diverted away from growth-related processes to prioritize survival mechanisms, as highlighted by the upregulated gene functions (Tucker and Koltunow, 2014; Du et al., 2022; Cai et al., 2025).

### 4.3 Protein interaction network reveals key functional modules driving drought adaptation in *Eucalyptus grandis*

The Protein-Protein Interaction (PPI) network (Figure 3) provides a visual representation of the functional relationships among the 472 robust DEGs in *E. grandis* subjected to drought stress, with node border color denoting STRING-DB module membership across four functionally enriched clusters: response to stimuli and stress (red), protein quality control and folding (yellow), plant growth, carbohydrate metabolism and biosynthesis (green), and DNA replication and repair (blue). The most prominent and densely interconnected module is the yellow cluster, enriched for protein quality control and folding, which constitutes the topological core of the network and is significantly enriched for ‘response to heat’ (GO:0009408, FDR $<1\times 10^{-17}$ ) and ‘protein folding’ (GO:0006457, FDR $<1\times 10^{-14}$ ); this module is dominated by strongly upregulated nodes, encompassing multiple *HSP70* and *SHSP20* isoforms, Heat Shock Factor (*HSF*) activators, the co-chaperone *BAG* (logFC=+4.22) , and J-domain-containing proteins (*JD*), and notably also includes *PIMT* (logFC=+1.80) , a protein-L-isoaspartate *O*-methyltransferase that directly repairs isomerized amino acids in damaged proteins, indicating that the drought response in *E. grandis* combines chaperone-mediated damage prevention with active enzymatic proteome repair, a two-pronged strategy for maintaining cellular homeostasis under prolonged water deficit (Wang et al., 2004; Kang et al., 2022; Zheng et al., 2025).

Also, PIMT is linked to a downregulated protein containing a WD40 repeats region (WDRR, logFC −1.81). This protein is connected to a highly coordinated cluster of other downregulated proteins essential for DNA replication, including multiple MCM DNA helicases (logFCs −1.64, −1.57, and −1.46), Sld5 (logFC −1.76), and a Replication Protein A (RPA) subunit (logFC −1.84). The coordinated downregulation of this group is a molecular signature of cell cycle arrest, a critical energy conservation strategy under drought. Since WD repeats are known to form scaffolds for protein complexes, the suppression of this specific protein likely helps arrange the shutdown of DNA replication (Çelik et al., 2023; Li et al., 2025). Then, the connection between the upregulated protein repair enzyme (PIMT) and this downregulated replication group visually represents a key strategic shift in the plant’s drought response of actively suppressing costly cell division while simultaneously boosting the maintenance and repair of existing cellular machinery to ensure survival (Pedroza-Garcia et al., 2016; Liu and Lang, 2020; Cadavid et al., 2023).

This central group of interacting proteins also revealed significant metabolic adjustments and osmoprotective strategies. The upregulation of an Inos-1-P-synth domain-containing protein (INO1, logFC +2.32), responsible for myo-inositol-1-phosphate synthesis, points to the accumulation of inositol-derived osmolytes. This finding is consistent with literature indicating that INO1 upregulation and subsequent inositol accumulation contribute to drought and salinity tolerance in various plant species by helping maintain cell turgor (Majumder et al., 2003; Baisakh et al., 2008; Gangwar et al., 2022). Similarly, trehalose 6-phosphate phosphatase (TPP, logFC +1.72) was shown as upregulated, suggesting the synthesis of trehalose, another key osmoprotectant and signaling molecule. Carbohydrate metabolism showed complex readjustments, with Starch synthase (SS, one instance at logFC +2.35) being upregulated, while Sucrose synthase (SUS, logFC −1.95) and a Glycosyl hydrolase 32 family member (GH32, logFC −3.19) were downregulated, indicating a significant redirection of carbon resources (Griffiths et al., 2025; Kong et al., 2021; Peng et al., 2015; Barbosa et al., 2023). These findings align with a conserved metabolic strategy in plants under abiotic stress, characterized by the accumulation of osmoprotectants and a significant redirection of carbon resources, a pattern where the upregulation of INO1 and the downregulation of growth-related genes like Sucrose Synthase (SUS) suggest a trade-off diverting carbon from growth to survival. Such a strategy is corroborated by recent studies; for instance, one of them (Yu et al., 2024) demonstrated in upland cotton (*Gossypium hirsutum*) that the positive regulation of GhIPS1-A, an ortholog of myo-inositol-1-phosphate synthase, is directly linked to maintaining fibre yield under drought, underscoring the critical role of the inositol pathway in preserving agronomic traits during stress. This focus on specific protective pathways is complemented by the findings of one of the works (Teshome et al., 2023) selected for this pipeline, which showed a general downregulation of metabolism and growth genes under drought, confirming a broader strategy of resource conservation that prioritizes survival mechanisms over growth, mirroring the specific gene expression changes presented in your data.

Beyond these central responses, other distinct clusters of interacting proteins highlighted other adaptive strategies. Enhanced antioxidant defense mechanisms were suggested by the upregulation of thioredoxin (TRX, logFC +2.62), crucial for redox homeostasis (Bashandy et al., 2010; König et al., 2012; Peng et al., 2015; da Fonseca-Pereira et al., 2019). Alterations in cell wall dynamics were implied by the differential expression of proteins such as a Pectin Methylesterase Inhibitor (Cheng et al., 2022) (PMEI, logFC −2.28), which influences cell wall rigidity, and various glycosyl hydrolases (*e.g.*, GH5, logFC −1.69; GH18, logFC +1.75) (Bian et al., 2021). Hormonal signaling adjustments also extended to auxin pathways, as evidenced by the downregulation of an auxin-responsive PIN protein (logFC −2.69) (Nam et al., 2023), consistent with stress-induced growth inhibition. In contrast, a protein containing an AAI domain (logFC +5.63), often associated with alpha-amylase inhibitors or stress responses, was strongly upregulated (Jiang et al., 2012; Zhou et al., 2016). Signaling and regulatory processes were also strongly represented within this major functional module. Several PPM-type phosphatase domain-containing proteins (PPM/PP2C, with consistently positive logFCs: +2.14, +2.32, +2.35, +2.67) were upregulated. These PP2C phosphatases are critical, often negative, regulators in abscisic acid (ABA) signaling, a cornerstone of plant drought response. Their upregulation in eucalyptus aligns with their documented involvement in stress tolerance across diverse plant systems. While the precise outcome of PP2C upregulation can be context-dependent, it signifies an active modulation of the ABA-mediated stress response (Fujii et al., 2009; Ma et al., 2009; dos Santos et al., 2014; Singh et al., 2015; Manohar et al., 2017; He et al., 2020). Other signaling components, including Glycosyltransferase 1 family proteins (GST1, logFCs +1.24, +2.18) (Ma et al., 2025), a putative UDP-glycosyltransferase (Peng et al., 2015; Liu et al., 2021) (UGT, logFC +2.82), a non-specific serine/threonine protein kinase (STK, logFC +1.26), and a tyrosine-protein phosphatase (PTP, logFC +1.65), were also differentially regulated, indicating broad involvement of phosphorylation cascades and conjugation pathways (Benešová et al., 2012; You and Chan, 2015; Wang et al., 2016).

Indications of significant shifts in secondary metabolism or hormone biosynthesis pathways were observed through the strong upregulation of several Fe(II)- and 2-oxoglutarate-dependent dioxygenases (Fe2OGD, logFCs +2.24, +3.20, +4.36). However, one member of this Fe2OGD family (logFC −3.03) and a Cytochrome P450 (CYP, logFC −3.45) were downregulated, suggesting specific modulations within these broad enzyme families (Brookbank et al., 2021; Lange et al., 2023). Furthermore, classic indicators of robust drought adaptation were prominently featured, most notably the very strong upregulation of a dehydrin (DHN, logFC +6.93), a protein well-known for its role in protecting cellular structures from dehydration damage (Graether and Boddington, 2014; Murray and Graether, 2022; Szlachtowska and Rurek, 2023). Also, a homeobox domain-containing protein (logFC +3.24), a generic type of transcription factor, was upregulated (Haak et al., 2017; Xie et al., 2021; Li et al., 2022). The upregulation of a DOG1 domain-containing protein (logFC +2.20), linked to ABA signaling and abiotic stress tolerance, further emphasized the plant’s comprehensive adaptive measures (Krüger et al., 2025).

A significant aspect of this network is the presence of numerous uncharacterized/hypothetical proteins (UNK) that showed substantial changes in expression levels (*e.g.*, with logFCs such as +5.66, +4.20, and −1.57) across these interconnected functional groups. These proteins did not present any recognizable domains or protein families. Such uncharacterized proteins represent a reservoir of potentially novel genes that are crucial for the drought response in *E. grandis* and are candidates for future functional characterization.

In summary, the PPI network analysis paints a comprehensive picture of a multifaceted and highly coordinated known response of *E. grandis* to drought conditions. The findings emphasize the critical roles of protein protection and quality control, osmotic adjustment through the synthesis of compatible solutes like inositol and trehalose, active modulation of ABA signaling pathways, and a strategic inhibition of growth. These primary responses are complemented by adjustments in redox homeostasis, cell wall modifications, altered secondary metabolism, and the induction of well-established stress-protective proteins like dehydrins.

Finally, beyond confirming known drought response pathways, this study illuminated a substantial number of uncharacterized proteins that demonstrated significant and robust differential expression. These novel candidates, embedded within the identified functional networks, represent a valuable untapped resource for future research. Their functional elucidation could unveil new mechanisms of drought tolerance and provide novel targets for genetic improvement. Consequently, the comprehensive dataset of robust DEGs and their interactive networks serves as a critical resource for the scientific community, paving the way for targeted functional genomics and the development of molecular markers for breeding drought-resilient *E. grandis* varieties.

Ultimately, this research provides critical insights into the molecular arsenal *E. grandis* deploys in response to drought, an increasingly pertinent challenge given the escalating climate crisis and its impact on global forestry and ecosystems like the Brazilian Cerrado. Understanding these complex adaptive mechanisms is paramount for developing sustainable forestry practices and ensuring the productivity and resilience of eucalyptus plantations in a changing world. The identified robust gene signatures and functional networks offer tangible pathways for future innovations in tree breeding and biotechnology, contributing to the development of forests better equipped to withstand future environmental adversities.

## Data Availability

The datasets presented in this study can be found in online repositories. The names of the repository/repositories and accession number(s) can be found below: https://github.com/jvtarss/euca2025.
